# Clinical Outcomes of Severe Rhinosinusitis Complicated with Cavernous Sinus Syndrome

**DOI:** 10.3390/jcm13082420

**Published:** 2024-04-21

**Authors:** Jin-Yi Lin, Chien-Lin Liu, Zheng-Yan Dai, Yu-Ting Li, Yung-An Tsou, Chia-Der Lin, Chih-Jaan Tai, Liang-Chun Shih

**Affiliations:** 1 Department of Otorhinolaryngology-Head and Neck Surgery, China Medical University Hospital, Taichung 404, Taiwan; karta5880477@gmail.com (J.-Y.L.); 037953@tool.caaumed.org.tw (C.-L.L.); 036864@tool.caaumed.org.tw (Z.-Y.D.); d22052121@gmail.com (Y.-A.T.); d6355@mail.cmuh.org.tw (C.-D.L.); edsam@seed.net.tw (C.-J.T.); 2 Department of Otorhinolaryngology-Head and Neck Surgery, Asia University Hospital, Taichung 404, Taiwan; 3 School of Medicine, China Medical University, Taichung 404, Taiwan

**Keywords:** rhinosinusitis, sphenoiditis, cavernous sinus syndrome, cavernous sinus thrombosis, septic cavernous sinus thrombosis, fungal infection, aspergillosis, diplopia, Horner syndrome, diabetes

## Abstract

**Background:** Various diseases involving the cavernous sinus can cause a condition called cavernous sinus syndrome (CSS), which is characterized by ophthalmoplegia or sensory deficits over the face resulting from the compression effect of internal structure. While tumor compression is the most reported cause of CSS, statistical data on CSS caused by infections are limited. Its risk factors, treatment methods, and clinical outcomes are not well-documented. **Methods:** In this retrospective study, we reviewed the data of patients admitted to a tertiary medical center from 2015 to 2022 with a diagnosis of acute and chronic sinusitis and at least one diagnostic code for CSS symptoms. We manually reviewed whether patients were involved in two or more of the following cranial nerves (CN): CN III, CN IV, CN V, or CN VI, or at least one of these nerves with a neuroimaging-confirmed lesion in the cavernous sinus. **Results:** Nine patients were diagnosed with rhinosinusitis-related CSS. The most common comorbidity was type 2 diabetes, and the most common clinical manifestations were diplopia and blurred vision. The sphenoid sinus was the most affected sinus. One patient expired due to a severe brain abscess infection without surgery. The remaining patients underwent functional endoscopic sinus surgery, and 50% of the pathology reports indicated fungal infections. *Staphylococcus* spp. was the most cultured bacteria, and Amoxycillin/Clavulanate was the most used antibiotic. Only four patients had total recovery during the follow-up one year later. **Conclusions:** CSS is a rare but serious complication of rhinosinusitis. Patients with diabetes and the elderly may be at a higher risk for this complication. Even after treatment, some patients may still have neurological symptoms.

## 1. Introduction

The cavernous sinus, located on either side of the sella turcica, plays a critical role in the venous drainage of the brain and eye, housing important structures such as the internal carotid artery, cranial nerves III (oculomotor), IV (trochlear), V1 (ophthalmic branch of the trigeminal), V2 (maxillary branch of the trigeminal), and VI (abducens), as well as sympathetic nerve fibers. These elements are encased within a dural fold, making the cavernous sinus a key site for neurological and vascular interactions.

Cavernous sinus syndrome (CSS) emerges when any of these contained structures are compromised, typically by compression, inflammation, or invasion. The resulting clinical manifestations can include ophthalmoplegia due to impairment of the cranial nerves responsible for eye movements and sensory deficits over the ophthalmic and maxillary regions, reflecting damage to the branches of the trigeminal nerve. Additional symptoms might include Horner’s syndrome from the disruption of the sympathetic pathway, decreased corneal reflex, and, in severe cases, altered mental status or visual loss if the condition progresses to affect the optic nerve or cause increased intracranial pressure. 

The pathophysiology behind CSS varies depending on the etiology. Tumors can physically compress the sinus or infiltrate its structures, leading to dysfunction. Infections, particularly those extending from nearby structures like the sinuses in rhinosinusitis, can lead to thrombophlebitis of the cavernous sinus (septic cavernous sinus thrombosis), an emergent condition requiring immediate attention. Autoimmune conditions and vascular diseases, such as carotid cavernous fistulas, can alter the flow within or damage the sinus, further contributing to symptomatology [1]. While there are relevant literature reviews, data specifically on cases caused by rhinosinusitis are limited, and there is no complete description of the associated causative factors and treatment guidelines. Therefore, it is crucial to investigate the risk features and effectiveness of medical or surgical treatment through case studies. 

## 2. Patients and Method

In this retrospective study, we reviewed the medical records of all patients admitted to a tertiary medical center in Taiwan from 2015 to 2022.

As there is no International Classification of Diseases (ICD) diagnostic code for CSS, hospitalization records were searched for any main diagnostic code (ICD10: J01 and J32 for acute and chronic sinusitis, respectively) or at least one diagnostic code for symptoms of CSS (such as G52.7–G52.8 Disorders of cranial nerves, G90.2 Horner’s syndrome, and H05 Disorders of the orbit). This screening method increases the chances of identifying potential cases of CSS. However, as many other diseases may also co-occur with these symptoms, further manual review and identification were completed to confirm that the disease was indeed CSS caused by rhinosinusitis. 

The inclusion criteria for patients in this study required evidence of the involvement of two or more of the following cranial nerves: CN III, CN IV, CN V (V1, V2), or CN VI, or at least one of these nerves with a neuroimaging-confirmed lesion in the cavernous sinus. The exclusion criteria were as follows: a diagnosis of stroke with imaging evidence, a history of a tumor with cranial involvement, a mucocele compression as indicated by imaging studies, a diagnosis of thyroid-associated ophthalmopathy, or orbital cellulitis without evidence of cavernous sinus involvement. 

The medical records were analyzed, and the following data were collected for statistical analysis: basic information (age, sex, and body mass index), common comorbidities (hypertension, diabetes mellitus, autoimmune diseases, and acquired immunodeficiency syndrome), clinical symptoms, imaging findings (number of involved sinuses on computed tomography (CT) scan and Lund–Mackay score), pathological reports, bacterial or fungal culture results, and treatment modalities such as surgical procedures and conservative management. The treatment outcomes of each case were followed up for one month, six months, and one year to assess residual symptoms and postoperative complications. 

Categorical variables were evaluated using Fisher’s exact test, while continuous variables were analyzed using one-way ANOVA via SPSS software (version 26, IBM, Armonk, NY, USA). Due to the small number of enrolled patients, a descriptive analysis without inferential statistics was performed.

Furthermore, we searched the PubMed database using the keywords “cavernous sinus syndrome”, “cavernous sinus thrombosis”, “septic cavernous sinus thrombosis” and included case series studies published in English with accessible full text from 2000 to 2022. We examined their abstracts and screened for relevant case series related to our topic. We found CSS caused by rhinosinusitis and compared these findings with our study results.

## 3. Results

**Figure 1:**
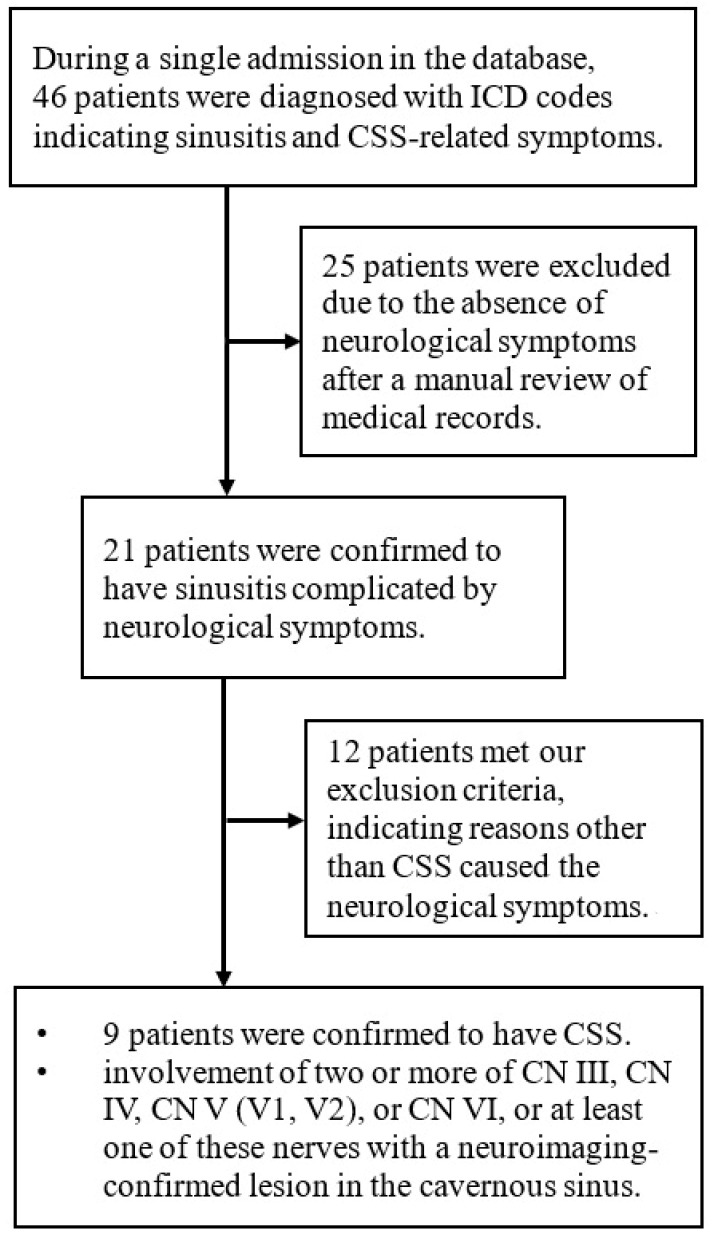
Flow diagram outlining the study design.

From 2015 to 2022, we identified 46 patients through ICD codes, and 25 of them were excluded after a manual review of their medical records, owing to them not relating to rhinosinusitis. Among the remaining 21 patients, 12 were excluded since their neurological symptoms were likely caused by stroke, intracranial tumors, mucoceles, thyroid-associated ophthalmopathy, or non-cavernous sinus-related orbital cellulitis (Figure 1). 

Of the patients included in the study, most patients did not undergo long-term follow-up in our ENT clinic before their surgery. Instead, they were seen in other departments, such as ophthalmology, neurology, or even emergency departments, due to neurological symptoms, leading to referrals for clinic assessments or hospital admissions. Specifically, two patients came through ophthalmology, three through neurology, and four through emergency departments. Consequently, the majority of patients had not previously been diagnosed with chronic sinusitis in an ENT setting, nor had they undergone related ENT surgeries. There were six male and three female individuals, with a mean age of 67.88 years at the time of diagnosis and a mean BMI of 25.8. Type 2 diabetes mellitus was the most common comorbidity (88%). The most frequent clinical presentations were diplopia (55%), ocular motility disturbance (55%), and blurred vision (55%); the other symptoms included ptosis, periorbital edema, headache, facial numbness, proptosis, fever, and blindness (Table 1). 

**Figure 2:**
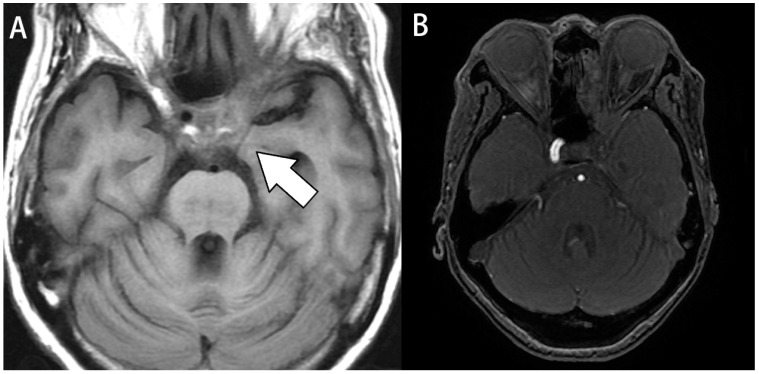
Image study of case 8. A 61-year-old woman with a history of diabetes presented with ptosis, double vision, extraocular muscle limitation, blurred vision, and fever. (**A**) Axial T1- weighted images. Fluid accumulated over the left cavernous sinus, causing luminal narrowing of the left carotid artery (white arrow). (**B**) Magnetic resonance angiography (MRA). MRA revealed occlusion in the left internal carotid artery.

**Figure 3:**
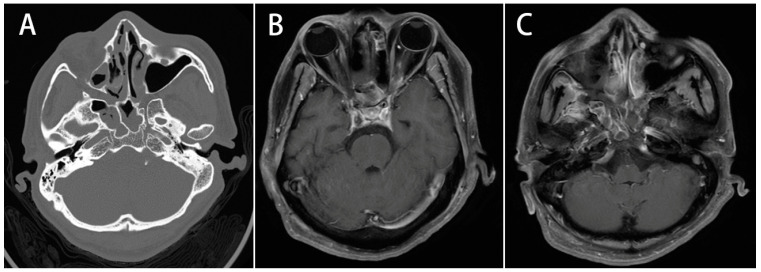
Image study of case 3. A 44-year-old man with a history of poorly controlled diabetes presented with eyelid swelling, blindness, alert mental status, mydriasis, fever, and headache. (**A**): Axial contrast-enhanced CT scan. CT scan revealed rhinosinusitis over bilateral sphenoid sinuses, complicated with subperiosteal empyema, bony wall erosion, and right orbital infection. (**B**,**C**): Axial T1-weighted MRI images. MRI showed bilateral bulging of the cavernous sinus and signal enhancement over cavernous sinus and skull base under T1-weighted images.

All patients were subjected to CT imaging examination regardless of whether they underwent surgery or not. Among them, six underwent magnetic resonance imaging (MRI) examination, and two underwent follow-up MRI after surgery. Some clinical features found on MRI or CT helped in the diagnosis of the disease, including asymmetric or bilateral bulging of the cavernous sinus, signal enhancement of lesions on T1-weighted images, luminal narrowing of the carotid artery, and erosion of the bony wall of the cavernous sinus (Figure 2 and Figure 3). The imaging studies confirmed that the most involved sinus was the sphenoid sinus (88%), followed by the ethmoid sinus (77%). A mean Lund–Mackay score was 10.55 for all patients. Detailed CT and MRI imaging data statistics are presented in Table 2.

One patient expired without surgical intervention due to a concomitant severe intracranial abscess, and there is no pathology report for this patient as he did not undergo a biopsy. The enduring eight patients underwent functional endoscopic sinus surgery (FESS). Nearly all patients had their surgery scheduled within a week of diagnosis confirmation. Pathology reports or culture results showed that 50% of the patients had fungal infections—two cases of *Aspergillus* infection, one of them also indicating invasive mucormycosis and another showed co-infection with *Candida albicans*, and two cases of infection by fungal hyphae found in tissue sections but without culture reports. *Staphylococcus* spp. (50%) were the most frequently cultured bacteria. Other pathogens included *Corynebacterium*, *Cutibacterium*, *Klebsiella pneumoniae*, *Pseudomonas aeruginosa*, and *Streptococcus* spp. The most utilized antibiotic during hospitalization was Amoxycillin/Clavulanate (44%). 

Regarding clinical outcomes, one patient expired during hospitalization (11%) due to central nervous system involvement, and among the patients who underwent surgical treatment, one patient did not return for a follow-up appointment after being discharged, five patients still had neurological symptoms 1 month after surgery (62.5%), and two patients fully recovered (25%). A follow-up 6 months after surgery showed that there was one patient who previously, without signs of recovery, experienced partial improvement and one patient who previously experienced partial improvement achieved full recovery of symptoms. During the one-year follow-up period after discharge, there was another patient who expired from central nervous system infection, while four fully recovered and two still had neurological symptoms. Patient’s inpatient antibiotic treatment, pathogen culture reports, and clinical outcomes are presented in Table 3.

## 4. Discussion

Cavernous sinus syndrome is one of the complications of local infection involving the adjacent orbits, sinonasal cavities, or facial soft tissues. Headache, orbital pain, ophthalmoplegia, and vision loss due to venous congestion within the retina raise clinical suspicion of cavernous sinus involvement [2]. We collected all sinusitis-associated CSS papers published in English on PubMed from 2000 to 2023 and discussed their case characteristics and clinical findings. The results are summarized in Table 4.

A review of small-scale studies showed that the majority of enrolled patients are elderly individuals, which is consistent with our study; however, there are still several case reports on young and pediatric patients (Table 4) [4,7,9,10].

Diabetes mellitus was the most common comorbidity (89%) in our study, which is consistent with the findings of previous studies [7,10,11]. This observation may be related to the immunocompromised state of patients with diabetes, especially those with poor disease control. A prospective study of 73 cases at a tertiary care center in northern India showed that the presence of diabetes was significantly associated with fungal CSS [12]. As for whether the use of medications affects the onset of the disease, such as topical or systemic steroids causing immunosuppression and, thereby, increasing the severity of the disease or the chances of infection, unfortunately, in our case series, there were no records of patients using relevant medications long-term to observe such phenomena. Previous systematic reviews indicated that the use of systemic steroids may increase the risk of acute exacerbations of chronic rhinosinusitis. However, whether this further increases the chance of CSS remains to be clarified [13]. Double-blind trials showed that the use of topical steroid nasal sprays in patients with chronic rhinosinusitis does not increase the risk of infection [14]. Still, further research is needed on medication use in immunocompromised patients.

Blurred vision was reported as one of the most observed eye-related symptoms (55%), and blindness was observed in one patient in our study, who was the only dead patient. Although the structure of the cavernous sinus does not include the optic nerve, which conveys visual information, the spread of infection can cause local inflammation and lead to associated complications. Thus, visual loss may be due to corneal ulceration, ophthalmic arterial occlusion, venous embolism, ischemia of the optic nerve, or intraorbital compression [5]. In our review of cases, all patients presented with unilateral symptoms, consistent with findings from other studies we reviewed. Bilateral neurological symptoms appear to be exceptionally rare. Our perspective is that while bilateral sinus involvement is common, the cavernous sinus is anatomically distinct from the nasal cavity and sinuses. It is not directly affected by the accumulation of pus in a consistent bilateral manner. Instead, abnormalities in the cavernous sinus may result from surrounding inflammatory reactions or pathogens carried by blood circulation. This process makes it challenging to ensure consistent bilateral involvement, hence our observation of predominantly unilateral lesions. Therefore, if a patient presents with bilateral neurological symptoms, other differential diagnoses must be carefully considered.

There are studies on the distribution of symptoms in larger samples of patients with CSS, but these studies did not specifically focus on patients with infections, and statistics have not been independently analyzed for patients with rhinosinusitis of different etiologies. Consequently, the most common symptoms of CSS may vary depending on the etiology [15].

Owing to the complexity of the cavernous sinus, imaging may not directly show obvious changes in the cavernous sinus in the early stages of the disease. Nevertheless, it can still help exclude certain diagnoses, such as stroke, which can also cause neurological defects. Computed tomography is often the first-line diagnostic tool because it can help identify rhinosinusitis and assess disease severity. Thus, all patients underwent CT scans during hospitalization in our study. However, MRI has an advantage in the evaluation of soft tissue structures such as the carotid artery and mucosa around the cavernous sinus. Therefore, some patients in our study who did not undergo MRI before surgery but still had neurological symptoms after surgery were scheduled for MRIs to obtain further information. Bhatkar et al. showed that CT scans had an overall sensitivity of 14.6% for achieving a final diagnosis, whereas MRI had an overall sensitivity of 70.7%, with a significant difference [16]. It is noteworthy that their study included patients with noninfectious CSS.

On account of the anatomical proximity of the sphenoid sinus to the cavernous sinus, the statistics showed a higher proportion of sphenoid sinus involvement than the other paranasal sinuses, as expected. Interestingly, in one patient from our study, there was no apparent involvement of the sphenoid sinus on imaging, but MRI revealed significant maxillary sinusitis and signal enhancement in the T1 flair phase, with contrast observed in the skull base bone extending from the maxillary sinus. Osteomyelitis secondary to maxillary sinusitis may extend along the skull base and involve the cavernous sinus or the surrounding tissues.

Previous reviews of the bacteriology of acute sphenoiditis have shown that *Streptococcus* is the most common organism associated with sphenoidal disease [6,17]; therefore, Amoxycillin/Clavulanate appears to be widely used as a first-line empiric antibiotic therapy. However, the occurrence of CSS suggests a more invasive infection that is commonly observed in immunocompromised patients. Therefore, in recent years, there have been increasing reports of CSS caused by co-infection with fungal pathogens. Thus, antifungal antibiotics should be considered if there is evidence of fungal infection [8,18,19,20].

A previous study indicated that a combination of antibiotics and FESS is the optimal treatment strategy for CSS caused by paranasal sinusitis [6]. The purpose of the surgery was to give drainage of the source of infection in the paranasal sinuses, including sphenoidectomy, ethmoidectomy, or maxillary antrostomy. The direct approach to cavernous sinus is difficult and can be risky because of the involvement of the internal carotid artery and numerous cranial nerves [21]. In our study, all patients underwent FESS with the navigation system except for one who expired due to multiple complications before the surgery. The post-operative clinical outcomes seem acceptable. No obvious surgical complications were noted after surgery.

It is worth noting that, upon review, we observed that the stage at which the disease was initially identified and its acute severity significantly influenced the duration of hospitalization. Patients admitted through the emergency department (cases 3, 7, 8, 9) appeared to have longer hospital stays compared to those admitted through outpatient services. Additionally, their antibiotic regimens were more complex. This may suggest the importance of early disease detection in outpatient settings.

In terms of the post-FESS one-month follow-up, three showed improvements but still had remaining neurological symptoms, such as extraocular movement limitation or blurred vision, and one showed no improvement in the first month after discharge from our ward. But, after 6 months of follow-up, two patients showed further improvement in their neurological symptoms, suggesting that neurological symptoms may take time to fully recover. One patient developed a recurrent infection and expired due to central nervous system involvement during the one-year follow-up, implicating the possibility of recurrent infections even after surgical treatment. However, based on available data, our treatment strategy prevented disease progression to some extent. 

Our study has certain limitations. Firstly, the absence of an ICD diagnosis code for CSS and the selection methods may have resulted in the possibility of missing some patients despite our efforts to obtain comprehensive data. The limited number of cases raises concerns about the overall credibility of the study. Nonetheless, we find some consistency with previous studies, offering a partial validation of our findings. Secondly, being a retrospective study, there is a potential for recall bias among the subjects during follow-up, which could impact our interpretation of the results. Inadequacies in medical record documentation and incomplete data can lead to the misinterpretation of essential information or make it difficult to review. For example, the patients’ unhealthy habits, such as smoking and alcohol intake, were not well documented and included in the manuscript. It is important to note that our research was conducted at a single tertiary medical center, which may restrict the generalizability of the results to other medical settings or regions. Furthermore, the treatment details from previous care at other regional hospitals or clinics cannot be retrieved completely from the medical record system. The patient population and treatment approaches in this particular hospital may not be representative of other healthcare settings. Additionally, as our study included only Asian individuals, the prevalence of pathogens may vary across different regions. 

In terms of future research directions, most of the anatomical imaging data were obtained after the onset of symptoms, which may influence the findings and overlook the opportunity to explore the intriguing topic of anatomically predicting disease onset. Obtaining longer-term imaging data from patients before the onset of symptoms may further advance the prediction of the disease.

## 5. Conclusions

Cavernous sinus syndrome is a serious complication of rhinosinusitis, and statistical analysis shows that elderly individuals and patients with diabetes may be at a high risk. If a patient with rhinosinusitis exhibits neurological symptoms in the orbital area and imaging studies confirm sphenoid sinus involvement, aggressive treatments, including antibiotics and surgical interventions, should be considered to prevent further risk of central nervous system involvement. Most CSS patients may have neurological symptoms after treatment and may take time to recover. Future research should focus on collecting large case data and longer-term imaging data to comprehensively explore the related risk factors, clinical manifestations, diagnosis, and different treatment strategies.

## Figures and Tables

**Table 1:** Basic data, comorbidity, and clinical signs of patients with CSS.

Case	Age/Sex	BMI	HTN	DM	Dyslipidemia	Other Underlying Conditions	Clinical Signs on Admission
Case 1	66 year/M	30.09	+	+	+	CAD, HBV, CRS s/p FESS, and Luc procedure 10 years ago	double vision, EOM limitation
Case 2	77 year/M	22.5	+	+	−	renal insufficiency	ptosis, eyelid swelling, mydriasis
Case 3	44 year/M	20.45	−	+	−	−	eyelid swelling, blindness, alert mental status, mydriasis, fever, headache
Case 4	73 year/F	29.37	+	−	−	OA knee, CAD	facial numbness
Case 5	79 year/M	31.36	+	+	-	Af, gout, CAD	double vision, EOM limitation
Case 6	72 year/M	23.42	−	+	−	−	double vision, blurred vision, headache
Case 7	74 year/M	19.4	−	+	+	−	ptosis, EOM limitation, eyelid swelling, blurred vision, fever
Case 8	61 year/F	27.3	−	+	−	−	ptosis, double vision, EOM limitation, blurred vision, fever
Case 9	65 year/F	29	+	+	−	CAD	ptosis, double vision, EOM limitation, facial numbness, eyelid swelling, blurred vision

BMI, body mass index; HTN, hypertension; DM, diabetes mellitus; M, male; F, female; CAD, coronary artery disease; HBV, hepatitis B; CRS, chronic rhinosinusitis; s/p, status post; Af, atrial fibrillation; OA, osteoarthritis. EOM, extraocular muscle.

**Table 2:** Sinus involvement and image feature on CT or MRI images of patients with CSS.

Case	Maxillary Sinus	Ethmoid Sinus	Frontal Sinus	Sphenoid Sinus	Lund–Mackay Score	Image Feature
Case 1	Bilateral	Bilateral	Bilateral	Bilateral	21	Status post bilateral FESS and left Luc procedure, Bilateral nasal polyposis, mucus retention, and mucosal swelling in sinuses
Case 2	Bilateral	Bilateral	Left	Bilateral	13	Extraconal infiltrative enhanced soft tissue density at medial superior aspect of left orbital cavity; secretion accumulation within the sinus antrum
Case 3	-	Bilateral	Right	Bilateral	14	Subperiosteal empyema, bony wall erosion, bilateral bulging of the cavernous sinus, and signal enhancement over cavernous sinus and skull base
Case 4	Bilateral	Left	-	Left	7	sinonasal polyposis
Case 5	Left	-	-	-	2	Left maxillary sinus heterogeneous opacification with calcification and mucoperiosteal thickening
Case 6	-	Bilateral	-	Bilateral	10	Expansion of the sphenoid sinus, with peripheral bony erosion, even destruction and disappearance of the clivus and the sellar floor
Case 7	Bilateral	Bilateral	Bilateral	Bilateral	12	Mucus retention with mucosa thickening in the bilateral maxillary, ethmoid, sphenoid, and frontal sinuses. Left cavernous sinus engorged
Case 8	-	Left	-	Left	3	Fluid accumulated over the left cavernous sinus; luminal narrowing of the left carotid artery
Case 9	-	Bilateral	Bilateral	Bilateral	13	Bilateral optic nerves and superior and oblique superior rectus muscles were swelling; middle and inferior turbinates in the left nasal cavity were enlarged; mucus retention with mucosa thickening in the bilateral ethmoid, frontal, and sphenoid sinuses
average	5 (55%)	7 (77%)	5 (55%)	8 (88%)	9.22	

**Table 3:** Antibiotic therapy, pathogen culture report, and clinical outcome of patient with CSS.

Case	Antibiotic Treatment	Pathology and Cultural Pathogen	Length of Hospital Stay	Outcome (1 Month Follow-up)	Outcome (6 Month Follow-up)	Outcome (1 Year Follow-up)
Case 1	Amoxycillin/Clavulanate	*Corynebacterium accolens*, *Cutibacterium acnes*, chronic paranasal sinusitis	5 days	Total recovery	Total recovery	Total recovery
Case 2	Teicoplanin, tazocin	-	7 days	Lost	Lost	Lost
Case 3	Fluconazole, tazocin, cefepime, vancomycin	-	14 days	Mortality	Mortality	Mortality
Case 4	Amoxycillin/Clavulanate	*Klebsiella pneumoniae*, *Streptococcus constellatus*, *Fusobacterium nucleatum*, Fungal sinusitis	3 days	Partial improvement	Partial improvement	Total recovery
Case 5	Amoxycillin/Clavulanate	*Citrobacter koseri*, *Staphylococcus epidermidis*, *Corynebacterium accolens*, *Cutibacterium avidum*, Fungal sinusitis	4 days	Partial improvement	Total recovery	Total recovery
Case 6	Amoxycillin/Clavulanate	chronic paranasal sinusitis	4 days	Total recovery	Total recovery	Total recovery
Case 7	Cefepime, vancomycin, voriconazole, tazocin	*Staphylococcus epidermidis*, *Candida albicans*, *Aspergillus*, chronic paranasal sinusitis	19 days	Partial improvement	Partial improvement	Partial improvement
Case 8	Ceftriaxone, clindamycin, cefepime, Fluconazole	*Pseudomonas aeruginosa*, *Staphylococcus epidermidis*, aspergillosis mucormycosis, chronic paranasal sinusitis	19 days	No improvement	Partial improvement	Mortality
Case 9	Ceftriaxone, vancomycin, rifampicin, levofloxacin, baktar	*Staphylococcus aureus* (MRSA), chronic paranasal sinusitis	21 days	Partial improvement	Partial improvement	Partial improvement

**Table 4:** Summary table of rhinosinusitis-related CSS case series from 2000 to 2023.

Author and Publish Date	Cases Number	Mean Age	Clinical Feature	Comorbidity	Sinus Involvement	Pathogen	Notes
Takahiko Yamanoi et al. [3]. June 2004	3	65.6	vision loss (3) ophthalmoplegia (3) exophthalmos (2) seizure (1)	not calculated	sphenoid (3)	*Aspergillus* (3)	This article mainly discussed successful cases of treating sphenoidal *Aspergillus* infection leading to cavernous sinus invasion with high-dose itraconazole therapy.
Huan-Wen Chen et al. [4]. March 2006	9	58.5	headache (5) diplopia (5) blurred vision (5) ptosis (4) fever (3) proptosis (2) facial numbness (2) photophobia (1)	DM (5) hematologic diseases (3)	ethmoid (6) sphenoid (4) maxillary (3) frontal (2)	Zygomycete (3) *Staphylococcus* (1) *Streptococcus* (1) fungus (1) *Aspergillus* (1) *Peptostreptococcus* (1)	The results of this study are consistent with our observations in terms of the patient population, including elderly individuals, patients with diabetes, and those with immunodeficiency, and also underscored the importance of fungal infections.
Franklin Lizé et al. [5]. January 2015	7	35.7	headache (7) fever (6) ptosis (3) chemosis (3) diplopia (3) proptosis (2) ophthalmoplegia (1) Horner’s syndrome (1) photophobia (1)	not calculated	sphenoid (7) ethmoid (3) maxillary (1) frontal (1)	*Streptococcus* spp. (3) *Staphylococcus aureus* (2) *Aspergillus fumigatus* (2) *Haemophilus influenzae* (1) *Serratia marcescens* (1) Buccal bacterial flora (1)	This study provided detailed statistics on the radiological characteristics of sinusitis-related CSS and the duration of anticoagulation therapy use.
Yun-Hu Wang et al. [6]. May 2017	8	30.8	headache (8) ophthalmoplegia (6) fever (5) ptosis (5) diplopia (4) photophobia (2) mydriasis (2)	not calculated	Sphenoid (8) Ethmoid (3) Maxillary (2)	*Streptococcus viridans* (2) *Mycobacterium avium* (1) MRSA (1) *Streptococcus intermedius* (1)	The clinical outcomes of this study were similar to ours, with comparable results in terms of disease characteristics, treatment, and effectiveness. It further emphasized the importance of anticoagulation therapy.
Che-Wei Hsu et al. [7]. October 2019	14	60.4	ophthalmoplegia (14) headache (13) ptosis (9) fever (9) chemosis (8) eyelid swelling (5) trigeminal hypesthesia (5) hemiparesis (5) eye pain (4) exophthalmos (4) decreased visual activity (3) Facial palsy (3) dysarthria (3) seizure (2) Horner’s syndrome (1)	DM (10) CRI (2) CHF (1) RA (1) COPD (1) NPC (1) liver cirrhosis (1)	sphenoid (13) ethmoid (11) maxillary (10) frontal (3)	*Aspergillus* spp. (6) *Staphylococcus* spp. (5) *Pseudomonas aeruginosa* (4) MRSA (2) *Acinetobacter baumannii* (2) *Citrobacter freundii* (2) CoNS (2) viridians streptococci (2) *Enterobacter faecalis* (1) *Veillonella dispar* (1) *Streptococcus constellatus* (1) *Fusobacterium nucleatum* (1) *Prevotella* spp. (1) Unknown fungus (1) *Citrobacter freundii* (1) *Propionibacterium* spp. (1) *Klebsiella pneumoniae* (1)	This study provided detailed pathogen culture results and specific antibiotic treatment regimens and evaluated the prognosis using the Modified Rankin Scale (MRS), discussing risk factors contributing to poor outcomes.
Han-wen Zhang et al. [8]. April 2020	4	58.7	visual disturbance (4) headache (4) facial paresthesia (2) proptosis (1) diplopia (1) ptosis (1) fever (1) pupils’ asymmetry (1)	DM (1) HTN (1) CAD (1)	sphenoid (4) ethmoid (1) maxillary (1)	*Aspergillus* species (4)	This study focused on aspergillosis-related CSS and included many cases of *Aspergillus* infections.

## Data Availability

The datasets used and/or analyzed during the current study are available from the corresponding author upon reasonable request.
